# Risk of colonoscopic post-polypectomy bleeding in patients on single antiplatelet therapy: systematic review with meta-analysis

**DOI:** 10.1007/s00464-021-08975-0

**Published:** 2022-01-13

**Authors:** Marco Valvano, Stefano Fabiani, Marco Magistroni, Antonio Mancusi, Salvatore Longo, Gianpiero Stefanelli, Filippo Vernia, Angelo Viscido, Silvio Romano, Giovanni Latella

**Affiliations:** 1 Gastroenterology Unit, Department of Life, Health and Environmental Sciences, University of L’Aquila, Piazzale Salvatore Tommasi 1, 67100 L’Aquila, Italy; 2 Cardiology Unit, Department of Life, Health and Environmental Sciences, University of L’Aquila, L’Aquila, Italy

**Keywords:** Aspirin, Platelet aggregation inhibitors, Antiplatelet agents, Haemorrhage, Intestinal polyps, Colonoscopy, Polypectomy, Post-polypectomy bleeding

## Abstract

**Background:**

It was not yet fully established whether the use of antiplatelet agents (APAs) is associated with an increased risk of colorectal post-polypectomy bleeding (PPB). Temporarily, discontinuation of APAs could reduce the risk of PPB, but at the same time, it could increase the risk of cardiovascular disease recurrence. This study aimed to assess the PPB risk in patients using APAs compared to patients without APAs or anticoagulant therapy who had undergone colonoscopy with polypectomy.

**Methods:**

A systematic electronic search of the literature was performed using PubMed/MEDLINE, Scopus, and CENTRAL, to assess the risk of bleeding in patients who do not interrupt single antiplatelet therapy (P2Y12 inhibitors or aspirin) and undergone colonoscopy with polypectomy.

**Results:**

Of 2417 identified articles, 8 articles (all of them were non-randomized studies of interventions (NRSI); no randomized controlled trials (RCT) were available on this topic) were selected for the meta-analysis, including 1620 patients on antiplatelet therapy and 13,321 controls. Uninterrupted APAs single therapy was associated with an increased risk of PPB compared to the control group (OR 2.31; CI 1.37–3.91). Patients on P2Y12i single therapy had a higher risk of both immediate (OR 4.43; CI 1.40–14.00) and delayed PPB (OR 10.80; CI 4.63–25.16) compared to the control group, while patients on aspirin single therapy may have a little to no difference increase in the number of both immediate and delayed PPB events.

**Conclusions:**

Uninterrupted single antiplatelet therapy may increase the risk of PPB, but the evidence is very uncertain. The risk may be higher in delayed PPB. However, in deciding to discontinue APAs before colonoscopy with polypectomy, the potential higher risk of major adverse cardiovascular events should always be assessed.

**Supplementary Information:**

The online version contains supplementary material available at 10.1007/s00464-021-08975-0.

Endoscopic techniques are becoming increasingly popular for both diagnostic and interventional procedures for gastrointestinal diseases. Given the high volume of these procedures, it is increasingly necessary to perform the endoscopic exam in a condition of safety for patients [1].

A meta-analysis including 14 studies, estimated that the overall pooled prevalence for mortality, perforation, and post-colonoscopy bleeding were 2.9/100,000 (95% confidence interval (CI) 1.1–5.5), 0.5/1,000 (95% CI 0.4–0.7), and 2.6/1000 (95% CI 1.7–3.7), respectively. This risk was higher in patients undergoing colonoscopic polypectomy, with a post-polypectomy bleeding (PPB) rate of 9.8 per 1000 polypectomies (95% CI 7.7–12.1) [2].

Furthermore, polypectomy is considered a high risk of bleeding procedure in particular in elderly patients [3, 4]. An observational study reported that age > 75 was independently associated with an increase of emergency department visit (OR 1.58; 95% CI 1.05–2.37) and hospitalization (OR 3.7; 95% CI 2.03–6.73) within 7 days of colonoscopy [5]. Taking into account the high mean age of patients who underwent an endoscopic procedure, it is common to find patients with many comorbidities [6] such as cardiovascular disease in antithrombotic treatment. It is estimated that 44.6% of over 70 s use aspirin among the U.S. population [7].

Antithrombotic therapy is used to reduce the risk of thrombotic/thromboembolic events in patients with several conditions such as atrial fibrillation (AF) and coronary artery disease (CAD); however, these drugs are linked to an increased risk of bleeding [8, 9]. Antithrombotic include anticoagulants or antiplatelet agents (APAs). The latter include aspirin and P2Y12 inhibitors (P2Y12i), such as clopidogrel, prasugrel, and ticagrelor which are commonly used to prevent thrombosis in patients who have had coronary stents, recent myocardial infarctions, peripheral stents for vascular disease, and cerebrovascular disease.

Many guidelines already exist on the management of APAs for the patient undergoing gastrointestinal endoscopy; Regarding the high-risk procedures, American guidelines (ASGE) suggest interrupting P2Y12i five days before the procedure in patients with low cardiovascular risk. Moreover, P2Y12i should be continued in patients at high risk of cardiovascular disease. The aspirin should never be interrupted [4]. While European (ESGE) guidelines suggest, in high-risk procedures, interrupting P2Y12i seven days before the procedure in patients with low cardiovascular risk; in patients with high cardiovascular risk is suggested to discuss strategy with a consultant interventional cardiologist. Patients on dual antiplatelet therapy should never interrupt the aspirin and consider temporary cessation of P2Y12i 6–12 months after drug-eluting stent insertion, or at least 1 month after bare metal stent insertion. However, the quality of this evidence ranges from moderate to low [3, 4].

For all these reasons, it is important to balance the post-polypectomy risk of bleeding and the risk of cardiovascular disease recurrence for the proper management of the suspensions APAs [10].

In particular, taking aspirin the risk of bleeding in a patient undergoing colonoscopy with polypectomy (with forceps, cold or hot snare) seems only slightly increased [3]; on the other hand, aspirin non-adherence or withdrawal is associated with a three-fold higher risk of major adverse cardiovascular events (OR 3.14; CI 1.75–5.61) [11]. Therefore, it seems reasonable to continue single antiplatelet therapy in patients undergoing colonoscopic polypectomy, in particular in patients with a high risk of cardiovascular disease [3]. To support this clinical evidence, it is necessary to assess PPB risk in patients on uninterrupted single antiplatelet therapy before colonoscopy with polypectomy.

We performed a systematic review with meta-analysis to assess the risk of bleeding in patients who did not interrupt single antiplatelet therapy (P2Y12i or aspirin) before colonoscopy with polypectomy.

## Materials and methods

### Study protocol

We reported a systematic review and meta-analysis according to the PRISMA guidelines [12] (Supplementary Table 1) using a predetermined protocol (PROSPERO n: CRD42020214769; October 2020).

A systematic electronic search for relevant publications (without language or date of publication restrictions) was performed by three investigators. The search included a combination of Medical Subject Headings (MeSH) and keywords (Supplementary Table 2).

Studies were identified using the following database: PubMed/MEDLINE, Scopus, and CENTRAL. Each of the relevant publication reference sections, and Google Scholar were also screened for other applicable publications. ClinicalTrial.gov was investigated to find unpublished completed trials.

Relevant abstracts were also screened. The last search was performed in January 2021.

We considered both randomized controlled trial and non-randomized studies (prospective and retrospective cohort studies, case–control studies, and analytical cross-sectional studies).

### Outcome of interest

The primary outcome of our meta-analysis was to assess the incidence of both immediate and delayed PPB in patients on APAs therapy undergoing colonoscopic polypectomy (expressed as dichotomous outcomes). We performed a subgroup analysis when it was possible, including:

- Risk of PPB (both immediate and delayed) in the P2Y12i group vs control.
- Risk of PPB (both immediate and delayed) in the aspirin group vs control.

Sensitivity analysis after the exclusion of studies not published as full-text and the studies with serious risk of bias assessed by ROBINS-I tool was performed [13].

The evidence produced in this meta-analysis was graded and presented according to the Grading of Recommendations Assessment, Development, and Evaluation (GRADE) system [14–16].

### Immediate post-polypectomy bleeding

Bleeding after polypectomy occurring at the time of colonoscopy or before discharge from the Endoscopy Unit.

### Delayed post-polypectomy bleeding

Rectal bleeding occurred the day after within 30 days after polypectomy.

### Selection of studies

Three authors (MV, AM, and SF) independently reviewed abstracts and manuscripts for eligibility. Conflicts were resolved by consensus, referring to the original articles. Studies were selected with the following criteria:

*Inclusion criteria*

1. Both randomized controlled trials (RCTs) and non-randomized studies of interventions (NRSI) with prospective or retrospective designs without language or date of publication restrictions.
2. Studies including patients on antiplatelet therapy (both P2Y12i and aspirin) undergoing colonoscopy with polypectomy.
3. Studies including a control group of patients without or discontinuing antithrombotic therapy.
4. Studies evaluating immediate PPB or complications after polypectomy.
5. Studies evaluating delayed PPB or complications after polypectomy.
6. Presented Odds ratio (OR), relative risk (RR), Hazard ratio (HR), or the number of events necessary to calculate these for the outcome of the interest rate.
7. When multiple publications from the same study or institution were available, the most recent publication has been used.

*Exclusion criteria*

1. Studies evaluating PPB or complications without an intervention group on APAs therapy (both P2Y12i and aspirin).
2. Studies evaluating PPB or complications with a control group with patients on antithrombotic therapy who were non-excludable.
3. Patients on dual antiplatelet therapy (DAPT) who were non-excludable from the intervention group.
4. Concomitant use of anticoagulant therapy (warfarin, direct oral anticoagulation, or heparin).
5. Patients underwent Endoscopic Mucosal Resection (EMR) or Endoscopic Sub-mucosal Dissection (ESD) who were non-excludable from both the intervention and control group.

### Data extraction and assessment of the risk of bias

Two reviewers (AM and SF) independently extracted the following data variables: title and reference details (first author, journal, year, country), study population characteristics (number of patients included in the study, gender and age, antiplatelet therapy, dosage, setting), outcome data (PPB, complication after polypectomy or death), polyp size and polypectomy technique.

All data were recorded independently by both literature reviewers in separate databases and were compared at the end of the reviewing process to limit selection bias. The database was then reviewed by a third person (MM) and any disparities were discussed and clarified with the consultation of the senior co-authors (AV and GL). Any conflicts were resolved by consensus, referring to the original articles.

The Authors of the eligible studies were contacted for additional information in the occurrence of the inconsistency of reported results during data extraction.

Two authors (MM and SF) independently assessed the risk of bias of included studies using the ROBINS-I tool [13]. Significant conflicts were resolved by consensus, re-evaluating the original articles, and if necessary, with the consultation of the senior co-authors (AV and GL).

Are considered as possible confounding domains relevant to all or most studies: polypectomy technique, polyps size, number of polyps per patient, location, morphology, histology, age, and comorbidities.

### Statistical analyses

Dichotomous outcomes were expressed as the OR with a 95% confidence interval (CI). The Odds Ratio for the individual study was combined using a random-effect model, with a fixed-effects model planned for non-significant heterogeneity (p > 0.10, I^2^ < 50%). The Mantel–Haenszel method was used to perform meta-analyses with the Review Manager software (Version 5.3. Copenhagen, Denmark: The Nordic Cochrane Centre, The Cochrane Collaboration, 2014). Heterogeneity was calculated using the χ^2^ test and I^2^ statistic defined by the Cochrane Handbook for Systematic Reviews [17].

We planned to examine publication bias using funnel plots for outcomes if data from 10 or more studies were available.

In this case, Egger’s regression test will be also performed for our primary analysis to assess for potential publication bias using the STATA/IC software Version v16.1 (2017, College Station, TX) [18].

Results will be considered statistically significant at the p < 0.05 level (if the 95% does not include the value of 1).

### Summary of findings and GRADE profile

We will present the main findings of the review concerning the certainty of the evidence, and magnitude of the effect of the interventions examined, in “Summary of findings” table, according to the GRADE [17, 19].

## Results

**Fig. 1:**
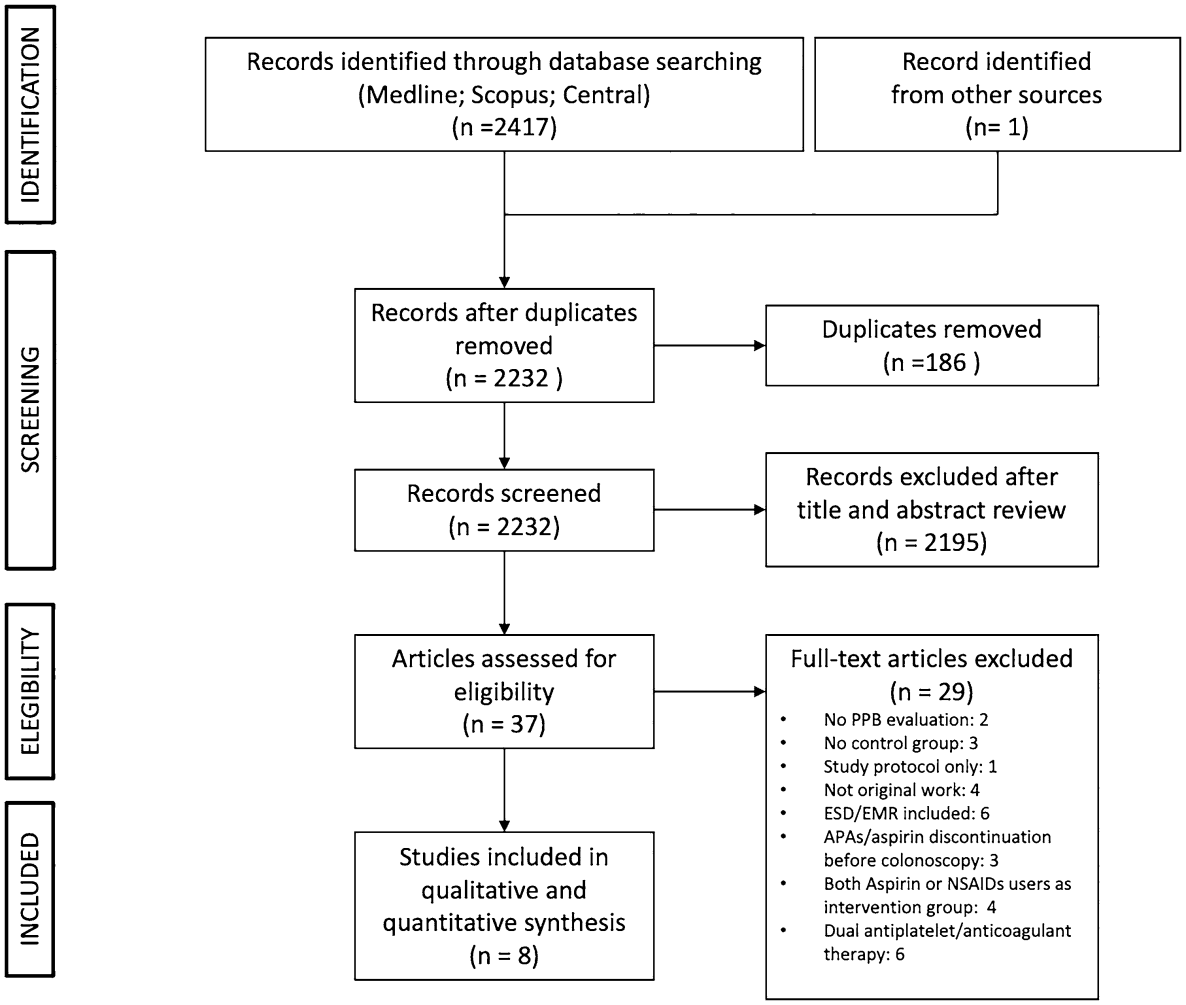
PRISMA flow diagram

Figure 1 shows the PRISMA flow diagram, including results of the literature search, as assessed by the three authors (MV, AM, and SF). We found 2417 articles, removing 186 duplicated records, excluding 2195 records based on their titles and abstracts. Among the 37 full texts assessed for eligibility, we included 8 articles (7 full-text and 1 abstract) for the quantitative synthesis, including 1,620 patients on antiplatelet therapy (P2Y12i or Aspirin) and 13,321 controls [20–27]. The characteristics of the eight selected studies are reported in Table 1 and in Supplementary Table 3. Definitions of clinical outcome measures set by individual studies are summarized in Table 2.

**Table 1:** Baseline characteristics of included studies

Study	Pop	Male %	Age^a,b^	Design	Setting	Uninterrupted antiplatelet agents	Definition of control group
Amato 2016 (Italy)	2692	54.3%	59 (± 12.1)^a^	NRSI (Prospective)	Multicentric; CRC screening	APAs	No treatment or APAs suspension for at least five days
Feagins 2013 (USA)	516	97%	62.4^a^	NRSI (Prospective)	VA hospital	APAs	No treatment
Grossman 2010 (USA)	3191	n.a	n.a	NRSI (Retrospective)	Endoscopic unit	Clopidogrel	No treatment
Hui 2004 (China)	1657	55.9%	64.4 (± 13)^a^	NRSI (Retrospective)	Endoscopic unit	Aspirin and Clopidogrel	No treatment
Kishida 2018 (Japan)	6382	70.6%	68 (17–96)^b^	NRSI (Retrospective)	Endoscopic unit	APAs	No treatment or antithrombotic suspension according to the JGES guidelines
Matsumoto 2018 (Japan)	1003	69.7%	n.a	NRSI (Retrospective)	Endoscopic unit	APAs	No treatment
Watanabe 2020 (Japan)	1050	72.1%	n.a	NRSI (Retrospective)	Endoscopic unit	APAs	No treatment
Yousfi 2004 (USA)	162	61.7%	72 (45–91)^b^	NRSI (Retrospective)	Multicentric; Endoscopic unit	Aspirin	No treatment

*NRSI* non-randomized study of intervention, *APAs* antiplatelet agents (both P2Y12i and aspirin), *CRC* colorectal cancer, *VA* veteran affairs, *JGES* Japan Gastroenterological Endoscopy Society

^a^Mean (± SD)

^b^Median (range)

**Table 2:** Clinical outcomes of included studies

Study	PPB definition	Immediate PPB definition	Delayed PPB definition	Intervention	PPB	Control	PPB	Severe bleeding (case/control)	Severe PPB definition
Amato 2016 (Italy)	n.a	Intra-procedural bleeding or before discharge	Bleeding ≤ 30 days after discharge	250	22^c^ (8.8%)	2431	83^c^ (3.4%)	n.a	Any bleeding leading to shock, blood transfusion, hospitalization, surgery, recurrent bleeding after endoscopic haemostasis and any perforation and death
Feagins 2013 (USA)	n.a	Intra-procedural bleeding that requires endoscopic treatment	Bleeding ≤ 30 days after polypectomy	146	9^a^ (6.1%)	178	7^a^ (3.9%)	0	Bleeding resulted in repeat colonoscopy, hospitalization, drop of haemoglobin by 2 g/dL or more, or blood transfusion
Grossman 2010 (USA)	n.a	Intra-procedural bleeding	Bleeding ≤ 30 days after polypectomy	70	6^c^ (8.6%)	2380	23^c^ (1%)	0	n.a
Hui 2004 (China)	n.a	Intra-procedural bleeding that requires endoscopic treatment	Bleeding ≤ 30 days after polypectomy require hospitalization	135	5 (3.7%)	1506	28 (1.9%)	n.a	Transfusion of 5 blood units or more. Angiographic or surgical intervention needed
Kishida 2018 (Japan)	Bleeding requiring endoscopic haemostasis (≤ 30 days)	n.a	n.a	687	4^d^ (0.6%)	5381	40^d^ (0.7%)	4/40	n.a
Matsumoto 2018 (Japan)	Bleeding requiring endoscopic intervention, open surgery or blood transfusion	n.a	n.a	68	1 (1.5%)	817	2 (0.2%)	0	n.a
Watanabe 2020 (Japan)	Rectal bleeding after polypectomy	n.a	n.a	205	8 (3.9%)	525	7 (1.3%)	n.a	n.a
Yousfi 2004 (USA)	Haemorrhage requiring transfusion, hospitalization, endoscopic intervention, angiography, or surgery	n.a	n.a	59	32^b^ (54.2%)	103	49^b^ (47.6%)	14/14	n.a

*na* not available

^a^Immediate

^b^Delayed

^c^Both immediate and delayed

^d^Severe bleeding

Six studies used a retrospective design [20–22, 25–27], while two used a prospective design [23, 24]. Six studies included both aspirin and P2Y12i users. Among these studies, five were separately analysed in a subgroup analysis to establish the PPB risk for each group [21, 23–25, 27].

One study included in our meta-analysis was a cohort study including only patients with significant PPB and a matched control group without complication at colonoscopy. As result, the incidence of PPB bleeding was higher than the other studies (54.2% vs 47.6% in APAs and control group, respectively) [20].

Three studies assessed the PPB rate in patients who underwent colonoscopic polypectomy with hot snare [27], cold snare [25], or both [26].

Uninterrupted APAs single therapy was associated with an increased risk of PPB compared to control group (5.4% vs 1.8%).

The risk of bias of the included studies assessed by the ROBINS-I is summarized in Supplementary Table 4; 7 studies had moderate risk of bias and 1 had serious risk.

Table 3 showed the main results of the review concerning the certainty of the evidence and magnitude of the effect of the interventions examined.

**Table 3:** GRADE profile

Certainty assessment							Summary of findings		Comments
Participants (studies) Follow-up	Risk of bias	Inconsistency	Indirectness	Imprecision	Publication bias	Overall certainty of evidence	Relative effect (95% CI)	Risk difference with PPB on single APAs/aspirin therapy	
*PPB on single antiplatelet therapy*									
14,941 (8 observational studies)	very serious^a^	serious^b^	not serious	not serious	none	⨁◯◯◯ Very low	OR 2.31 (1.37 to 3.91)	23 more per 1.000 (from 6 to 49 more)	The APAs may increase the risk of PPB, but the evidence is very uncertain
*PPB on P2Y12i therapy*									
6512 (5 observational studies)	very serious^a^	not serious	not serious	serious^d^	none	⨁◯◯◯ Very low	OR 5.29 (2.99 to 9.37)	75 more per 1.000 (from 36 to 136 more)	The P2Y12i may increase the risk of PPB, but the evidence is very uncertain
*PPB on aspirin therapy*									
6313 (6 observational studies)	serious^c^	not serious	not serious	not serious	none	⨁⨁⨁◯ Moderate	OR 1.87 (1.32 to 2.65)	26 more per 1.000 (from 10 to 48 more)	The aspirin probably results in a slight increase in PPB
*Immediate PPB on P2Y12i therapy*									
5124 (3 observational studies)	very serious^a^	serious^g^	not serious	very serious^d^	none	⨁◯◯◯ Very low	OR 4.43 (1.40 to 14.00)	52 more per 1.000 (from 6 to 173 more)	The P2Y12i may increase immediate PPB, but the evidence is very uncertain
*Immediate PPB on aspirin therapy*									
2940 (2 observational studies)	serious^c^	not serious	not serious	serious^f^	none	⨁⨁◯◯ Low	OR 1.43 (0.78 to 2.64)	12 more per 1.000 (from 6 fewer to 43 more)	The aspirin may result in little to no difference in immediate PPB
*Delayed PPB on P2Y12i therapy*									
4919 (2 observational studies)	very serious^a^	not serious	not serious	very serious^d^	none	⨁◯◯◯ Very low	OR 10.80 (4.63 to 25.16)	59 more per 1.000 (from 23 to 134 more)	The P2Y12i may increase delayed PPB, but the evidence is very uncertain
*Delayed PPB on aspirin therapy*									
2805 (2 observational studies)	serious^c^	serious^e^	not serious	very serious^d^	none	⨁◯◯◯ Very low	OR 2.50 (0.63 to 9.87)	36 more per 1.000 (from 9 fewer to 181 more)	The aspirin may increase delayed PPB, but the evidence is very uncertain

*CI* confidence interval, *OR* odds ratio

^a^A large study (Grossman 2010) have a serious risk of bias in three domains (Bias due to confounding, Bias due to missing data, bias in measurement of outcomes)

^b^Moderate heterogeneity. It is widely explainable considering the different drugs in the intervention group

^c^All the studies included have at least one domine at moderate risk of bias. No serious or critical risk biases were detected

^d^Wide confidence intervals and small sample size

^e^High heterogeneity due to difference in included patients. Yousfi 2004 included only patients with PPB in the case group, with a control group identified among patients matched for age, gender, and cardiovascular morbidity

^f^Very small sample size

^g^Moderate heterogeneity due to a large single abstract

### Description of excluded studies

The reasons for the exclusion of 29 studies that were not included in this review are summarized in Supplementary Table 5. Among these studies, 2 did not evaluate PPB; 3 did not include a control group without antithrombotic therapy; 1 was a study protocol; 4 were not original works; 6 included patients undergoing both ESD and EMR; 3 discontinued P2Y12i or aspirin before the colonoscopy; 4 included both aspirin or NSAIDs users as intervention group; 6 included patients on DAPT or anticoagulant therapy.

### Overall immediate and delayed post-polypectomy bleeding

Eight studies assessed the PPB risk in patients on single antiplatelet therapy (P2Y12i or aspirin) [20–27]. Out of 1620 patients on single APAs therapy, 181 patients were on uninterrupted P2Y12i single therapy, 751 were on uninterrupted aspirin single therapy, 688 were on uninterrupted APAs single therapy (which APAs was not specified).

**Fig. 2:**
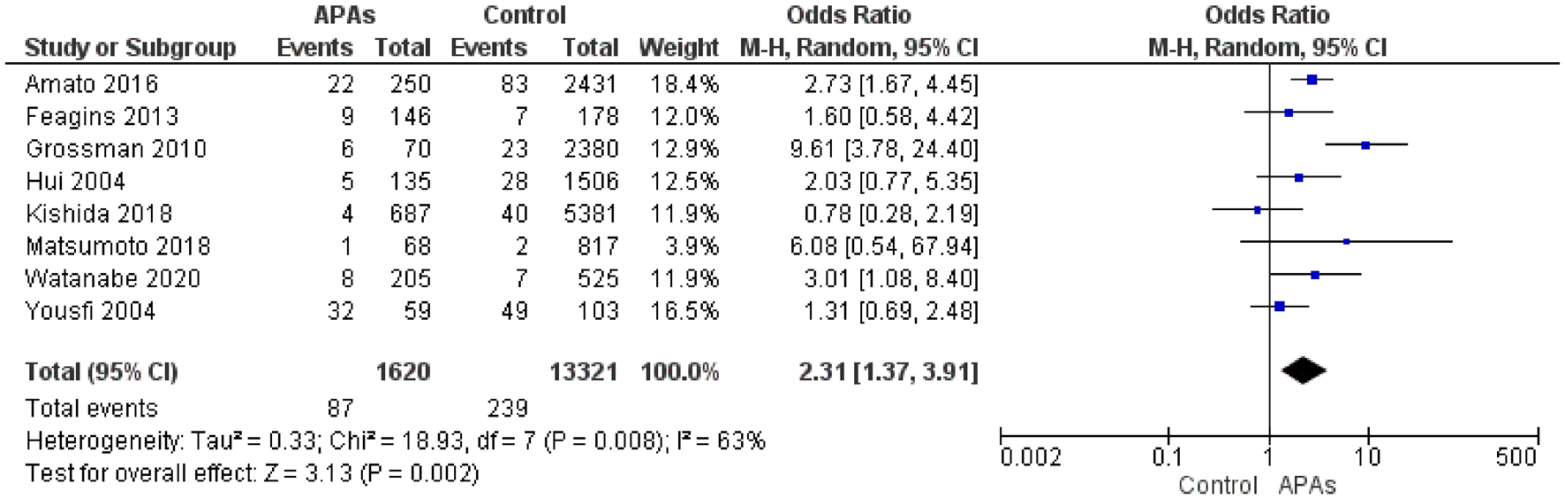
Overall PPB in APAs single therapy

Uninterrupted APAs single therapy was associated with an increased risk of PPB compared to control group (OR 2.31; CI 1.37–3.91) (Fig. 2). The heterogeneity found in this analysis is widely explainable in the subgroup analysis of aspirin, clopidogrel, and other P2Y12i users.

**Fig. 3:**
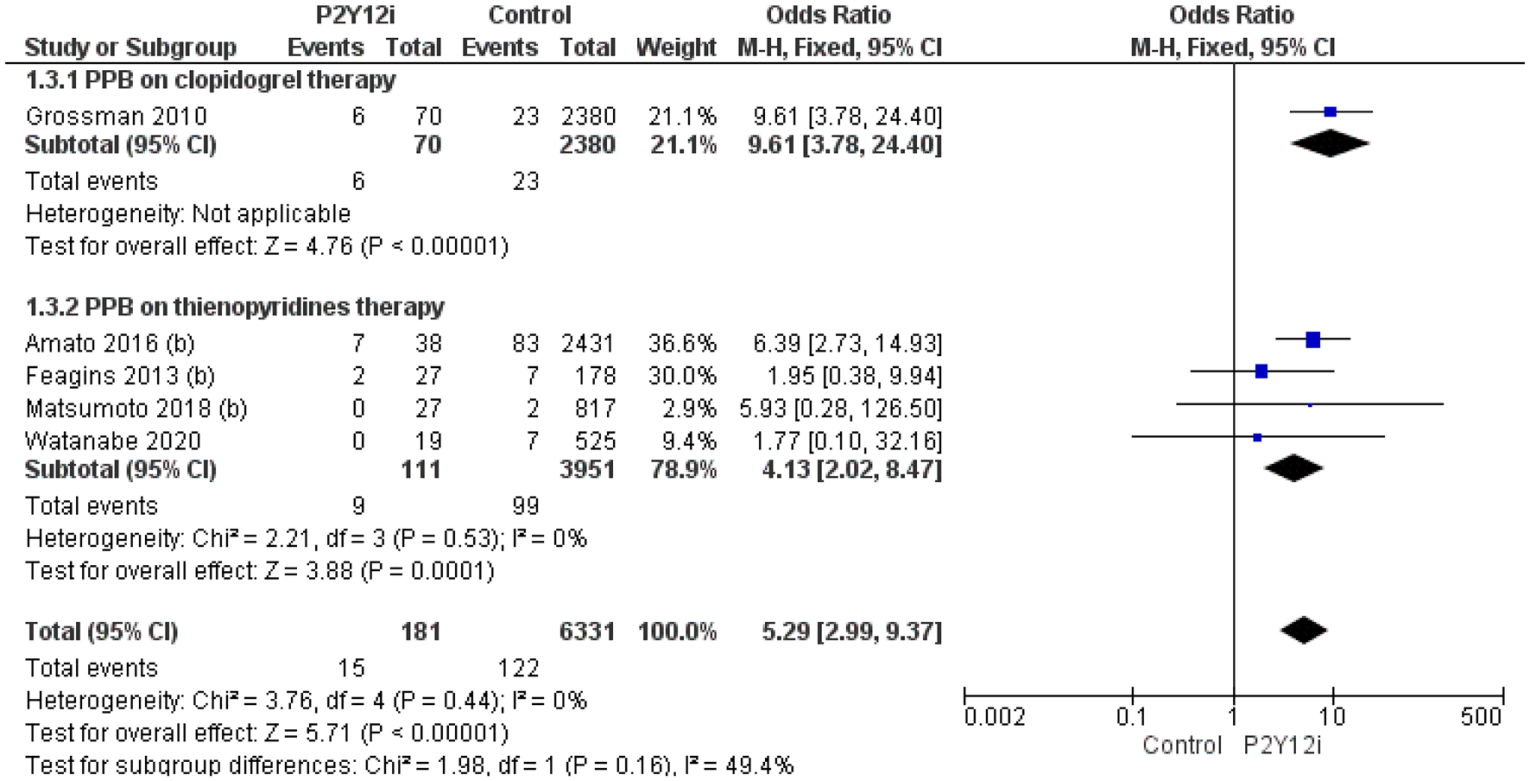
Overall PPB in P2Y12i single therapy

**Fig. 4:**
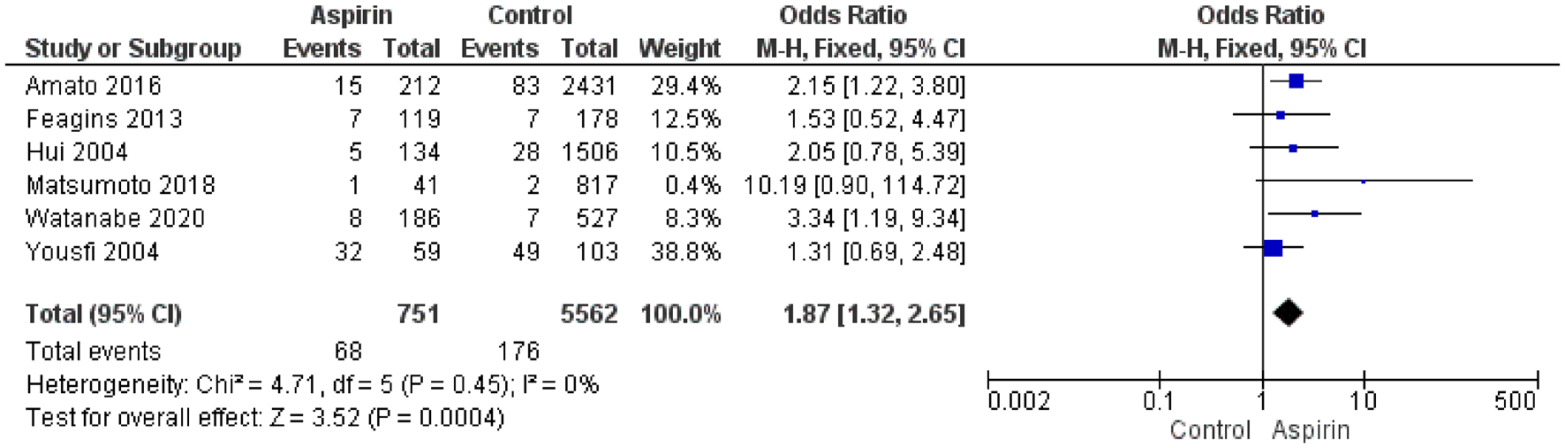
Overall PPB in aspirin single therapy

Uninterrupted clopidogrel and other P2Y12i were associated with a higher risk of PPB (OR 5.29; CI 2.99–9.379) than uninterrupted aspirin (OR 1.87; CI 1.32–2.65) compared to control (Figs. 3, 4).

### Immediate post-polypectomy bleeding

Three studies (2 full-text and one abstract) [22–24] assessed the immediate PPB risk in patients on uninterrupted P2Y12i therapy. Uninterrupted P2Y12i was associated with an increased risk of PPB compared to control group (OR 4.43; CI 1.40–14.00) although it was higher in clopidogrel users than in the other P2Y12i user group (OR 13.28 and OR 2.59, respectively) (Supplementary Fig. 1).

Two studies evaluated the immediate PPB risk in patients on uninterrupted aspirin therapy. [23, 24] Among uninterrupted aspirin group, there were no significant differences in the number of immediate PPB bleeding events compared to the control group (OR 1.43; CI 0.78–2.64) (Supplementary Fig. 2).

### Delayed post-polypectomy bleeding

Four studies evaluated the delayed PPB risk in patients on uninterrupted APAs. Two studies for P2Y12i (one full-text and one abstract) [22, 24] and two for aspirin [20, 24]. Uninterrupted P2Y12i single therapy was associated with an increased risk of PPB compared to control, while there were no significant differences among uninterrupted aspirin group compared to the control group (OR 10.80; CI 4.63–25.16 and OR 2.50; CI 0.63–9.87, respectively) (Supplementary Figs. 3, 4).

### Sensitivity analysis and publication bias

Sensitivity analysis was conducted excluding the abstract that was the only study with a serious risk of bias. Among the full text, the overall PPB prevalence was higher among patients on APAs therapy compared to controls with a lower heterogeneity (OR 1.83; CI 1.35–2.49) (Supplementary Fig. 5).

We performed a sensitivity analysis excluding the only study which included only patients with PPB in the case group, with a control group identified among patients matched for age, gender, and cardiovascular morbidity. In this study, the prevalence of PPB was 50% [20]. The pooled OR of PPB in APAs users was 2.59 (CI 1.45–4.63) (Supplementary Fig. 6).

The funnel plot and the Egger’s regression test for publication bias weren’t performed because only 8 studies were included.

## Discussion

The results of our meta-analysis showed that patients on single antiplatelet therapy such as P2Y12i or aspirin had a 2.31-fold higher risk of bleeding compared to control (CI 1.37–3.91).

This risk appeared higher among patients on clopidogrel therapy. It is important to underline that, only an abstract assessed the risk of PPB among patients on clopidogrel.

Among patients on aspirin single therapy, the overall PPB risk was 2.04-fold higher (CI 1.48–2.80), although there was no difference in both immediate (OR 1.43; CI 0.78–2.64) and delayed PPB (OR 2.50; CI 0.63–9.87) compared to the control group. It is important to underline that these data were available only in two studies. Therefore, both these subgroup analyses involved a suboptimal sample size.

Moreover, patients on P2Y12i single therapy had a higher risk of both immediate and delayed PPB (immediate OR 4.43; CI 1.40–14.00; delayed OR 10.80; CI 4.63–25.16).

The data concerning the size of lesions, localization, and resection techniques in patients on APAs were not extractable in relation to the outcomes of our meta-analysis. On the other hand, the great majority of polyps evaluated in the included studies were < 10 mm; therefore, based on the above data, our result is less easily generalizable for polyps > 10 mm. Among the overall population of the studies included: 13,841 patients underwent hot snare polypectomy, 5882 patients underwent cold forceps polypectomy, 3332 patients underwent cold snare polypectomy, 746 patients underwent hot biopsy polypectomy; in three studies some techniques were counted in pairs, 532 patients underwent hot + cold snare polypectomy, 3057 patients underwent cold snare + cold forceps polypectomy. Moreover, 19,635 patients that underwent polypectomy had polyps < 10 mm and 4206 patients that underwent polypectomy had polyps > 10 mm. Data concerning which drug the patients were taking related to the technique or the size of the polyps were not extractable, making it impossible to compare these data and obtain the outcome of interest.

Our meta-analysis has some limitations. First, we included only observational studies because RCTs assessing the PPB risk among patients on single antiplatelet therapy are not currently available.

Second, only one abstract assessed the PPB risk (both delayed and immediate) among patients on clopidogrel, and not any performed a sub-analysis among the other P2Y12i. Furthermore, PPB among patients in single antiplatelet therapy was the main outcome in only two included studies [20, 22].

A recent RCT, published by Chan and colleagues, evaluated the risk of PPB with uninterrupted clopidogrel therapy vs placebo, taken until the day of colonoscopy. The results showed that a slightly larger proportion of patients continuing clopidogrel developed delayed (3.8% vs 3.6%) and immediate (8.5% vs 5.5%) post-polypectomy bleeding, although this difference was not statistically significant [28]. However, only 7.7% of the polyps included in this RCT, were ≥ 10 mm in size, so the RCT is strongly underpowered for this subgroup analysis, and the generalizability of the conclusion for polyps > 10 mm is limited [29, 30]. Moreover, about 80% of patients were on DAPT and these data are hardly generalizable to a group of patients who are not on dual antiplatelet therapy. It is important to underline that PPB is rarely life threatening, whereas a thrombotic event caused by clopidogrel interruption can be harmful. Therefore, any discussion about the reduction in the risk of PPB is of secondary importance compared to cardiovascular thrombotic events caused by interruption of antiplatelet therapy [29, 30].

Few meta-analyses evaluated the colonoscopic post-polypectomy bleeding in patients on antiplatelet therapy [31–34]. However, none of these assessed the risk of PPB in single APAs. Two of these meta-analyses evaluated the PPB risk in patients exposed to both aspirin and NSAIDs [31, 33]. Moreover, Pigò et al. included patients who underwent colorectal polypectomy with snare, ESD, or EMR. Colorectal ESD on APAs, except for aspirin alone, were independent risk factors for delayed bleeding (OR 4.04; CI 1.44–11.30) [3, 35]. ASGE guidelines recommend discontinuation of thienopyridines at least 5 to 7 days before high-risk endoscopic procedure or switching to aspirin monotherapy which may be continued safely in the peri-endoscopic period [4]. Two well-conducted meta-analyses assessed the pooled relative risk ratio of colonoscopic PPB in patients who continued clopidogrel therapy; however, they both included patients on an uninterrupted single APAs therapy or DAPT [32, 34].

On the other hand, although patients on APAs therapy have an established increased risk of PPB, aspirin non-adherence or withdrawal is associated with a three-fold higher risk of major adverse cardiovascular events (OR 3.14; 1.75–5.61) [11]. A U.S. observational study including 2197 cases of ischemic stroke identified through hospital discharge records, reported that 5.2% of strokes occurred within 60 days of an antithrombotic medication withdrawal [36]. Therefore, it is essential to balance the PPB risk of endoscopic polypectomy and the risk of major adverse cardiovascular events due to discontinuation of therapy.

In our meta-analysis, we analysed only patients in single antiplatelet therapy. These inclusion criteria are crucial for the APAs management before the colonoscopy with polypectomy. It is important to underline that patients on DAPT should suspend P2Y12i agents 7 days before the endoscopy and continue aspirin if they have low thrombotic risk, and liaise with a cardiologist about the risk/benefit of discontinuing P2Y12i in patients at high thrombotic risk [3].

Our result showed a moderate increase of PPB in patients with uninterrupted antiplatelet therapy. Although a moderate heterogeneity in our main outcome, these data appeared solid and the heterogeneity is widely explainable with the different PPB risk observed for the various antiplatelet agents, as shown in the subgroup analysis. Moreover, after the exclusion of the abstract (with a serious risk of bias) the pooled PPB risk in patients with uninterrupted antiplatelet agents remained higher compared to the control group, despite the slightest heterogeneity (OR 1.51; CI 1.03–2.22).

It is important to underline that no death was observed in all the studies included.

The risk of major adverse cardiovascular events in patients who discontinue aspirin single therapy is greater than the risk of delayed PPB in patients who continue this treatment.

Despite this, a U.S. survey showed that less than half of the endoscopy units routinely continue aspirin before colonoscopies [37]. Another German survey, regarding the interruption of clopidogrel and/or dual antiplatelet therapy, demonstrated that in this setting the decision has an individual basis because the current guidelines on endoscopic procedures in patients under clopidogrel/dual antiplatelet therapy are mainly based on expert opinion and supported by only weak evidence [38].

One of the possible causes of the scarcity of these data is given by the few therapeutic indications present in the current cardiological guidelines about P2Y12i single therapy [39, 40].

It is extremely important to produce more evidence and strongest data about PPB in patients on uninterrupted single antiplatelet therapy. In particular, there is a lack of RCTs assessing the increase in the risk of PPB among patients on single APAs therapy compared with patients who withdrawal the antiplatelet therapy. This setting would reflect the scenario for the management of APAs therapy in which the clinical decision is made.

In conclusion, uninterrupted single antiplatelet therapy may increase the risk of PPB, but the evidence is very uncertain.

Concerning P2Y12i, the guidelines suggest that the temporary interruption of this therapy should be carefully evaluated, considering the potential higher risk of major adverse cardiovascular events; however, P2Y12i may increase the risk of PPB, but the evidence is very uncertain.

Uninterrupted single aspirin therapy probably results in a slight increase of PPB when compared with control.

It is important to underline that, aspirin withdrawal results in high risk of major adverse cardiovascular events, thus it should be continued before the colonoscopic polypectomy.

Therefore, both the risk of endoscopic post-polypectomy bleeding and the risk of major adverse cardiovascular events should be assessed on a case-by-case basis, assessing both the degree of thrombotic risk and the degree of bleeding risk in the individual patient with the discontinuation of single antiplatelet therapy. However, to produce more clear and solid clinical evidence, RCTs including patients on single APAs therapy compared with patients who withdraw the antiplatelet therapy are needed.

## Supplementary Information

Below is the link to the electronic supplementary material.

Supplementary file1 (DOCX 110 kb)
