# Supplementary material for: Risk of colonoscopic post-polypectomy bleeding in patients on single antiplatelet therapy: systematic review with meta-analysis
Source: Surg Endosc. 2022 Jan 13;36(4):2258–70. doi: 10.1007/s00464-021-08975-0 (PMC8921031; doi:10.1007/s00464-021-08975-0)
Supplement: Supplementary file 1 — Supplementary file1 (DOCX 110 kb) [file 464_2021_8975_MOESM1_ESM.docx]

**Supplementary table 1: PRISMA 2020 Checklist**

| **Section and Topic** | **Item #** | **Checklist item** | **Location where item is reported** |
| --- | --- | --- | --- |
| **TITLE** | | |  |
| Title | 1 | Identify the report as a systematic review. | **Title page** |
| **ABSTRACT** | | |  |
| Abstract | 2 | See the PRISMA 2020 for Abstracts checklist. | **Abstract** |
| **INTRODUCTION** | | |  |
| Rationale | 3 | Describe the rationale for the review in the context of existing knowledge. | **Introduction** |
| Objectives | 4 | Provide an explicit statement of the objective(s) or question(s) the review addresses. | **Introduction** |
| **METHODS** | | |  |
| Eligibility criteria | 5 | Specify the inclusion and exclusion criteria for the review and how studies were grouped for the syntheses. | **Methods**:   - Inclusion criteria - Exclusion criteria - Statistical analyses |
| Information sources | 6 | Specify all databases, registers, websites, organizations, reference lists and other sources searched or consulted to identify studies. Specify the date when each source was last searched or consulted. | **Methods**:   - study protocol |
| Search strategy | 7 | Present the full search strategies for all databases, registers and websites, including any filters and limits used. | **Supplementary table and figure**:   - Supplementary table 2 |
| Selection process | 8 | Specify the methods used to decide whether a study met the inclusion criteria of the review, including how many reviewers screened each record and each report retrieved, whether they worked independently, and if applicable, details of automation tools used in the process. | **Methods**:   - Selection of studies |
| Data collection process | 9 | Specify the methods used to collect data from reports, including how many reviewers collected data from each report, whether they worked independently, any processes for obtaining or confirming data from study investigators, and if applicable, details of automation tools used in the process. | **Methods**:   - Data extraction and assessment of studies quality |
| Data items | 10a | List and define all outcomes for which data were sought. Specify whether all results that were compatible with each outcome domain in each study were sought (e.g. for all measures, time points, analyses), and if not, the methods used to decide which results to collect. | **Methods**:   - Outcome of interest: Immediate post polypectomy bleeding; Delayed post polypectomy bleeding |
|  | 10b | List and define all other variables for which data were sought (e.g. participant and intervention characteristics, funding sources). Describe any assumptions made about any missing or unclear information. | **Methods**:   - Outcome of interest; Immediate post polypectomy bleeding; Delayed post polypectomy bleeding. - Inclusion criteria - Exclusion criteria |
| Study risk of bias assessment | 11 | Specify the methods used to assess risk of bias in the included studies, including details of the tool(s) used, how many reviewers assessed each study and whether they worked independently, and if applicable, details of automation tools used in the process. | **Methods:**   - Data extraction and assessment of risk of bias |
| Effect measures | 12 | Specify for each outcome the effect measure(s) (e.g. risk ratio, mean difference) used in the synthesis or presentation of results. | **Methods:**   - Statistical analyses |
| Synthesis methods | 13a | Describe the processes used to decide which studies were eligible for each synthesis (e.g. tabulating the study intervention characteristics and comparing against the planned groups for each synthesis (item #5)). | **Methods:**   - Selection of studies - Inclusion criteria - Exclusion criteria |
|  | 13b | Describe any methods required to prepare the data for presentation or synthesis, such as handling of missing summary statistics, or data conversions. | **Methods:**   - Statistical analyses |
|  | 13c | Describe any methods used to tabulate or visually display results of individual studies and syntheses. | **Methods:**   - Summary of findings and GRADE profile |
|  | 13d | Describe any methods used to synthesize results and provide a rationale for the choice(s). If meta-analysis was performed, describe the model(s), method(s) to identify the presence and extent of statistical heterogeneity, and software package(s) used. | **Methods:**   - Statistical analyses |
|  | 13e | Describe any methods used to explore possible causes of heterogeneity among study results (e.g. subgroup analysis, meta-regression). | **Methods:**   - Outcome of interest |
|  | 13f | Describe any sensitivity analyses conducted to assess robustness of the synthesized results. | **Methods:**   - Outcome of interest |
| Reporting bias assessment | 14 | Describe any methods used to assess risk of bias due to missing results in a synthesis (arising from reporting biases). | **Methods**:   - Data extraction and assessment of the risk of bias |
| Certainty assessment | 15 | Describe any methods used to assess certainty (or confidence) in the body of evidence for an outcome. | **Methods:**   - Summary of findings and GRADE profile |
| **RESULTS** | | |  |
| Study selection | 16a | Describe the results of the search and selection process, from the number of records identified in the search to the number of studies included in the review, ideally using a flow diagram. | **Results**  **Figure 1:** PRISMA flow diagram |
|  | 16b | Cite studies that might appear to meet the inclusion criteria, but which were excluded, and explain why they were excluded. | **Results:**   - Description of excluded studies   **Supplementary table and figure**:   - Supplementary table 6 |
| Study characteristics | 17 | Cite each included study and present its characteristics. | **Results**  **Table 1-2** |
| Risk of bias in studies | 18 | Present assessments of risk of bias for each included study. | **Results:**   - Sensitivity analysis and publication bias   **Supplementary table and figure**:   - Supplementary table 4 |
| Results of individual studies | 19 | For all outcomes, present, for each study: (a) summary statistics for each group (where appropriate) and (b) an effect estimate and its precision (e.g. confidence/credible interval), ideally using structured tables or plots. | **Results**:   - Overall immediate and delayed post polypectomy bleeding in patients in single antiplatelet therapy - Immediate post polypectomy bleeding in patients in single antiplatelet therapy - Delayed post polypectomy bleeding in patients in single antiplatelet therapy; - **Figures 2, 3, 4**   **Supplementary table and figure**:   - Supplementary figure 1-4 |
| Results of syntheses | 20a | For each synthesis, briefly summarise the characteristics and risk of bias among contributing studies. | **Results:**   - Sensitivity analysis and publication bias   **Supplementary table and figure**:   - Supplementary table 4 |
|  | 20b | Present results of all statistical syntheses conducted. If meta-analysis was done, present for each the summary estimate and its precision (e.g. confidence/credible interval) and measures of statistical heterogeneity. If comparing groups, describe the direction of the effect. | **Results**:   - Overall immediate and delayed post polypectomy bleeding in patients in single antiplatelet therapy - Immediate post polypectomy bleeding in patients in single antiplatelet therapy - Delayed post polypectomy bleeding in patients in single antiplatelet therapy;   **Figures 2, 3, 4**  **Supplementary table and figure**:   - Supplementary table 5 |
|  | 20c | Present results of all investigations of possible causes of heterogeneity among study results. | **Results**   - Overall immediate and delayed post polypectomy bleeding in patients in single antiplatelet therapy - Immediate post polypectomy bleeding in patients in single antiplatelet therapy - Delayed post polypectomy bleeding in patients in single antiplatelet therapy); - Figures 2, 3, 4; - Supplementary Figures 1 - 4 |
|  | 20d | Present results of all sensitivity analyses conducted to assess the robustness of the synthesized results. | **Results**   - Sensitivity analysis and publication bias   **Supplementary table and figure**:   - Supplementary figure 5-6 |
| Reporting biases | 21 | Present assessments of risk of bias due to missing results (arising from reporting biases) for each synthesis assessed. | **Results**:   - Sensitivity analysis and publication bias. |
| Certainty of evidence | 22 | Present assessments of certainty (or confidence) in the body of evidence for each outcome assessed. | **Supplementary table and figure**:   - Supplementary table 5 |
| **DISCUSSION** | | |  |
| Discussion | 23a | Provide a general interpretation of the results in the context of other evidence. | **Discussion** |
|  | 23b | Discuss any limitations of the evidence included in the review. | **Discussion** |
|  | 23c | Discuss any limitations of the review processes used. | **Discussion** |
|  | 23d | Discuss implications of the results for practice, policy, and future research. | **Discussion** |
| **OTHER INFORMATION** | | |  |
| Registration and protocol | 24a | Provide registration information for the review, including register name and registration number, or state that the review was not registered. | **Methods**:   - Study protocol |
|  | 24b | Indicate where the review protocol can be accessed, or state that a protocol was not prepared. | **Methods**:  Study protocol |
|  | 24c | Describe and explain any amendments to information provided at registration or in the protocol. | **Not performed** |
| Support | 25 | Describe sources of financial or non-financial support for the review, and the role of the funders or sponsors in the review. | **Title page - Conflict of interest** |
| Competing interests | 26 | Declare any competing interests of review authors. | **Title page** |
| Availability of data, code and other materials | 27 | Report which of the following are publicly available and where they can be found: template data collection forms; data extracted from included studies; data used for all analyses; analytic code; any other materials used in the review. | **Available on request by contacting the corresponding author.** |

**Supplementary table 2: Research strategy**

| ("polypectomies"[All Fields] OR "polypectomy"[All Fields] OR "colonoscop*"[All Fields])  AND  ("aspirin"[MeSH Terms] OR "aspirin"[All Fields] OR "aspirins"[All Fields] OR "aspirin s"[All Fields] OR "aspirine"[All Fields] OR "acetylsalicylic Acid"[All Fields] OR ("clopidogrel"[MeSH Terms] OR "clopidogrel"[All Fields] OR "clopidogrel s"[All Fields]) OR ("prasugrel hydrochloride"[MeSH Terms] OR ("prasugrel"[All Fields] AND "hydrochloride"[All Fields]) OR "prasugrel hydrochloride"[All Fields] OR "prasugrel"[All Fields] OR "prasugrel s"[All Fields]) OR ("ticagrelor"[MeSH Terms] OR "ticagrelor"[All Fields]) OR ("thienopyridin"[All Fields] OR "thienopyridine"[Supplementary Concept] OR "thienopyridine"[All Fields] OR "thienopyridines"[MeSH Terms] OR "thienopyridines"[All Fields]) OR "antiplatelet agen*"[All Fields])  AND  ("bleedings"[All Fields] OR "hemorrhage"[MeSH Terms] OR "hemorrhage"[All Fields] OR "bleed"[All Fields] OR "bleeding"[All Fields] OR "bleeds"[All Fields] OR "post polypectomy bleeding"[All Fields] OR ("ppb"[All Fields]))  No filter or limits were applied.  CLINICAL TRIAL.GOV: Post-polypectomy bleeding |
| --- |

**Supplementary table 3: Resection technique and polyps characteristics of included studies**

| **Study** | **Resection Technique n (%)** | **Polyp Size, n (%)** |
| --- | --- | --- |
| Amato 2016 (Italy) | Hot snare (2471, 47,8%), cold snare (717, 13,8%)  and cold forceps (1621, 31,3%) | 4323 (83.5%) < 10 mm;  855 (16.5%) ≥ 10 mm |
| Feagins 2013 (USA) | Hot snare (515, 29,7%), cold snare (75, 4,3%) and cold forceps (1174, 67,8%) | 1636 (94.4%) < 10 mm;  96 (5,6%) ≥ 10 mm |
| Grossman 2010 (USA) | n.a. | n.a. |
| Hui 2004 (China) | Hot biopsy (746, 45%), snare (532, 32%), combined hot biopsy and snare (363, 22%) | Mean: 9,96 mm |
| Kishida 2018 (Japan) | Hot snare (9805, 76,2%), cold snare and cold forceps (3057, 23,8%) | 9916 (77%) < 10 mm  2946 (23%) ≥ 10 mm |
| Matsumoto 2018 (Japan) | Cold snare (2540, 97.3%), cold forceps (39, 2.7%) | 2579 (100%) < 10 mm |
| Watanabe 2020 (Japan) | Hot snare (1050, 100%) | 756 (72%) < 10 mm  294 (28%) ≥ 10 mm |
| Yousfi 2004 (USA) | Hot forceps and snare (86%), cold forceps and snare (7%) | 425 (96,6%) ≤ 10 mm  15 (3,4%) > 10 mm |

**Supplementary table 4: ROBINS-I**

| Reference | Confounding | Selection | Classification of intervention | Deviation from intended intervention | Missing data | Measurement of outcomes | Reported result | Overall |
| --- | --- | --- | --- | --- | --- | --- | --- | --- |
| Amato 2016 | **Moderate** | **Low** | **Moderate** | **Low** | **Low** | **Moderate** | **Low** | **Moderate** |
| Feagins 2013 | **Moderate** | **Low** | **Moderate** | **Low** | **Low** | **Moderate** | **Low** | **Moderate** |
| Grossman 2010 | **Serious** | **Low** | **Moderate** | **Low** | **Serious** | **Serious** | **Low** | **Serious** |
| Hui 2004 | **Moderate** | **Low** | **Moderate** | **Low** | **Moderate** | **Low** | **Low** | **Moderate** |
| Kishida 2018 | **Low** | **Low** | **Moderate** | **Low** | **Moderate** | **Low** | **Low** | **Moderate** |
| Matsumoto 2018 | **Moderate** | **Low** | **Moderate** | **Low** | **Moderate** | **Low** | **Low** | **Moderate** |
| Watanabe 2020 | **Moderate** | **Low** | **Moderate** | **Low** | **Moderate** | **Low** | **Low** | **Moderate** |
| Yousfi 2004 | **Moderate** | **Low** | **Moderate** | **Low** | **Low** | **Moderate** | **Low** | **Moderate** |

- **Low risk of bias**: “the study is comparable to well-performed randomized trial with regard to this domain.”
- **Moderate risk of bias**: “the study is sound for a non-randomized study with regard to this domain but cannot be considered comparable to a well-performed randomized trial.”
- **Serious risk of bias**: “the study has some important problems in this domain.”
- **Critical risk of bias**: “the study is too problematic in this domain to provide any useful evidence on the effects of intervention.”

**Overall risk of bias:** The result has an overall risk of bias at least as the more severe judgment. [17]

**Supplementary table 5: Excluded studies**

| **Study** | **Reasons for exclusion** |
| --- | --- |
| Nakajima H, at al. 1997 | No polypectomy included (no PPB evaluation) |
| Sorbi D, et al. 2000 | No control group |
| Ket S, et al. 2019 | Study protocol |
| Yabe K, et al. 2020 | Data not extractable; unknow number of patients on APAs and aspirin therapy; Patients on anticoagulant therapy included |
| Arimoto J, et al. 2019 | Only ESD performed |
| Radaelli F, et al. 2019 | Not original work |
| Sonneveld MJ, et al. 2019 | Not original work |
| Chan FKL, et al. 2019 | Data not extractable; Patients on dual antiplatelet therapy included |
| Lin D, et al. 2018 | No control group |
| Arimoto J, et al. 2018 | Only ESD performed |
| Shibuya T, et al 2017 | Data not extractable; Patients on dual antiplatelet therapy; Patients on anticoagulant therapy included |
| Ninomiya Y, et al. 2015 | Only ESD performed |
| Beppu K, et al. 2014 | APAs and aspirin discontinuation before colonoscopy |
| Choung BS, et al. 2014 | APAs and aspirin discontinuation before colonoscopy |
| Inoue T, et al. 2014 | Data not extractable; Patients on anticoagulant therapy |
| Burgess NG, et al. 2013 | Only EMR included |
| Moon HS, et al. 2014 | Data not extractable; EMR included; Patients on NSAIDs included as intervention group |
| Wu X-R, et al. 2013 | Data not extractable; Patients on dual antiplatelet therapy included |
| Manocha D, et al. 2012 | Data not extractable; Patients on NSAIDs included as intervention group |
| Pan A, et al. 2012 | Data not extractable; Patients on dual antiplatelet therapy included; Patients on anticoagulant therapy included |
| Feagins LA, et al. 2011 | Data not extractable; Patients on NSAIDs included in intervention group vs Patients on DAPT included in intervention group |
| Metz AJ, et al. 2011 | Data not extractable; EMR included; APAs and aspirin discontinuation before colonoscopy |
| Singh M, et al. 2010 | Data not extractable; Patients on NSAIDs included as intervention group; Patients on Aspirin and anticoagulant therapy included in both case and control group. |
| Argüello Viudez L. 2009 | Not original work |
| Sawhney MS, et al. 2008 | Data not extractable; Aspirin discontinuation before colonoscopy |
| Komatsu T, et al. 2005 | Polypectomy not performed (No PPB evaluation) |
| Friedland S, et al. 2009 | No control group |
| Lichtenstein D, et al. 2019 | Not original work |
| Shiffman, et al 1994 | Data not extractable; Patients on NSAIDs included as intervention group |

**Supplementary figure 1: Immediate PPB in P2Y12i users**

**
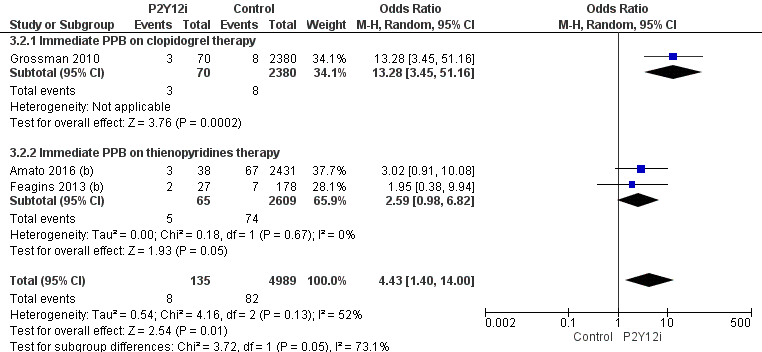
**

**Supplementary figure 2: Immediate PPB in aspirin users**

*
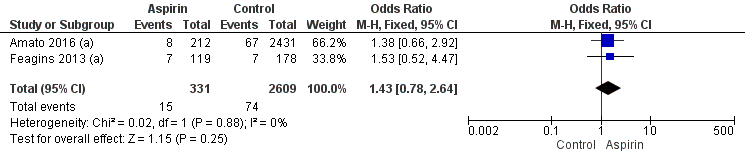
*

**Supplementary figure 3: Delayed PPB in P2Y12i users**


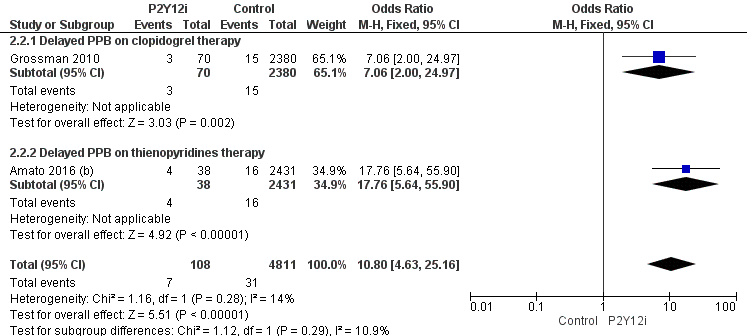


**Supplementary figure 4: Delayed PPB in aspirin users**


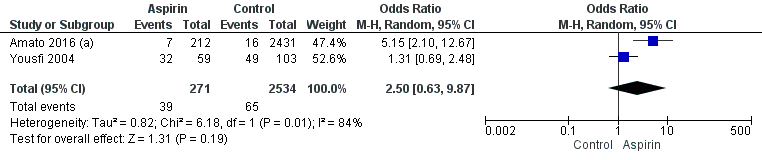


**Supplementary figure 5: Sensitivity analysis PPB full text/low to moderate risk of bias**


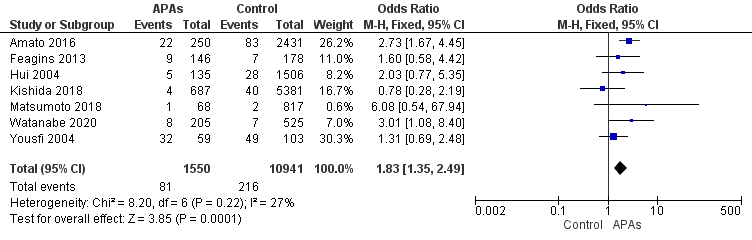


**Supplementary figure 6: Sensitivity analysis PPB 2**

**
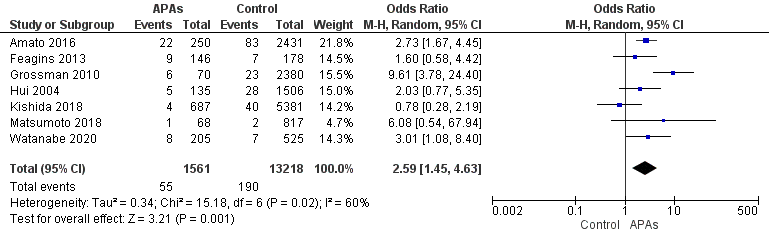
**
